# Mental-health profiling with person-centred analysis: A study of adolescents in Sweden

**DOI:** 10.1177/14034948231158850

**Published:** 2023-03-24

**Authors:** Charli Eriksson, Håkan Stattin

**Affiliations:** 1 Department of Learning, Informatic, Management and Ethics, Karolinska Institute, Sweden; 2 Department of Public Health, University of Stockholm, Sweden; 3 Department of Psychology, University of Uppsala, Sweden

**Keywords:** mental health, psychological symptoms, psychosomatic symptoms, adolescents, cluster analysis, dual-factor model

## Abstract

**Background:**

Studies that have examined time trends in adolescents’ mental health have frequently been based on analyses of adolescents’ psychosomatic symptoms. Adolescents’ reports of psychosomatic complaints need to be seen in the light of their overall evaluations of their health. The objectives of this study were to apply a person-centred approach to identifying adolescents’ mental-health profiles based on evaluations of their overall health and psychosomatic complaints. The relationships between these mental-health profiles and indicators of positive mental health and psychological and social-adjustment problems are examined.

**Methods:**

A dual-factor approach was used for nationally representative adolescent samples and examined adolescents’ self-rated psychosomatic complaints and perceived overall health simultaneously. Cluster analyses of data from the Swedish Health Behaviour in School-aged Children (HBSC) 2017/18 survey, including 3222 children aged 11, 13 and 15, were used to identify mental-health profiles.

**Results:**

Four mental-health profiles were identified by cluster analyses in all age groups. The profiles showed good construct validity in relation to mental well-being, life satisfaction and self-esteem. The poorest psychological adjustment was found among the adolescents with high levels of psychosomatic symptoms together with low levels of perceived overall health. Adolescents with high levels of psychosomatic symptoms only or with low levels of overall health only showed considerably better psychological adjustment.

**Conclusions:**

**Cluster analyses identified distinct, valid and consistent mental-health profiles based on differing levels of perceived health and psychosomatic complaints. The dual-factor model in population health research may increase our potential to understand the mental health of adolescents better.**

## Introduction

There are reasons to be concerned about the high prevalence of mental-health complaints among adolescents and the increases in symptoms over the last two decades [1–4]. But adolescents’ reports of their mental-health complaints need to be seen in the light of their overall evaluations of their health. These two perspectives integrate information from different self-perceptions of symptoms and of overall health. The need to differentiate between the traditional one-dimensional view of mental health and a two-dimensional one has been given prominence since the introduction of the dual-factor model in 2001 [5,6]. The dual-factor model gives a more complete description of the mental health of different population groups because it integrates two interrelated yet distinct constructs for assessing mental health and allows for the possibility that an increase in one is not necessarily associated with a decrease in the other. The dual-factor model of mental health uses two dimensions of mental health simultaneously: one dimension concerning mental illness or psychopathology (subjective complaints to psychiatric diseases) and a dimension concerning well-being (subjective well-being and health). A recent scoping review [5] found empirical support for the dual-factor model, that is, two related factors fitted the data in several studies. Moreover, identification of distinct subgroups within the dual-factor model using measures of these two perspectives on mental health also contributes to greater in-depth understanding of the distribution of mental health. The present study focuses on nationally representative samples of adolescents, with subjective health complaints (psychosomatic) and self-rated overall health (SRH) as its two dimensions. The study differs from previous dual-factor studies in its employment of a non-clinical symptom checklist rather than clinical diagnosis.

SRH is based on individuals’ perceptions and evaluation of their health overall. SRH can be distinguished from more specific health constructs in that it captures an overall conception of health. SRH, as it is operationalised, covers a continuum of health states, ranging from negative to positive depending on the overall perception of the state of the human body and mind [7].

Subjective health complaints are reported perceptions of adolescents with different psychosomatic symptoms, frequently occurring together, which provide important indicators of mental health and well-being. A high prevalence of complaints among adolescents, higher among girls than boys and showing an increasing time trend in certain countries, was reported in the Nordic region between 1993–1994 and 2013–2014 [8].

### Current study

The aim of the current study was to apply a person-centred approach to identifying adolescents’ mental-health profiles based on their health complaints and evaluations of perceived overall health. The study investigated cluster profiles among school-aged children and their relationships with indicators of positive mental health, perceived social support and school-related factors [9].

First, based on the current literature, we expected to find two profiles indicating poorer mental health among adolescents: high levels of psychosomatic symptoms only and high levels of both symptoms and health complaints. Second, the mental-health profiles were expected to differ on levels of positive mental health and with respect to social support and school-related factors, which should validate the constructs. We analysed the profiles across ages by clustering data on 11-, 13- and 15-year-olds. In these analyses, sex is important, since the gender gap in mental health in adolescence is ubiquitous cross-culturally, with girls having worse mental health [10].

## Methods

### Data material

The Health Behaviour in School-aged Children (HBSC) study is a cross-national research investigation into the health and well-being of adolescents across Europe and the USA conducted in collaboration with the World Health Organization (WHO) Regional Office for Europe [11]. The HBSC has been conducting surveys of young people every four years since 1983–1984, with an increasing number of countries participating in each survey cycle. Data are collected through school-based surveys using a standard methodology detailed in the HBSC international study protocol, which ensures that the sample is representative of all groups in the age range. The study is organised and developed by a network of HBSC national teams that include researchers based in a variety of academic and public health institutions. A total of 227,441 young people from 45 countries took part in the 2017–2018 survey.

The present study is part of our Nordic research collaboration on positive mental health among school-aged children [12]. The analyses are based on Swedish HBSC data from the 2017–2018 survey, which covered students in grades 5, 7 and 9 (ages 11, 13 and 15). Statistics Sweden performed the data collections. Two-step cluster sampling was used for each grade. First, a random, nationally representative sample of schools was drawn, and thereafter one class in each of the schools that had agreed to participate was randomly selected. Of the 450 schools that were included in grade 9, 213 (47%) agreed to participate [13] – a low response rate at school level that was due to the requirement for school anonymity. Statistics Sweden could not contact schools directly to remind them of the survey. In total, 4288 students participated, corresponding to 89% of the students in the schools that agreed to take part. The numbers of students by age were 1181 aged 11, 1452 aged 13 and 1655 aged 15.

### Measures

Clustering variables included two measures. The HBSC Symptom Checklist (HBSC-SCL) [11] has the stem question, ‘In the last 6 months: how often have you experienced. . . ?’ followed by eight symptoms: headache, stomachache, backache, feeling low, irritability or bad temper, feeling nervous, difficulties in getting to sleep and feeling dizzy. The response categories are 1=‘rarely or never’, 2=‘about every month’, 3=‘about every week’, 4=‘more than once a week’ and 5=‘about every day’. Alpha reliability was 0.81 for all ages aggregated.

Self-rated overall health was measured with the single item ‘Would you say your health is . . .?’ Participants were asked to rate their overall health by choosing one of the four response categories: 1=‘poor’, 2=‘fair’, 3=‘good’ and 4=‘excellent’ [11].

With regard to the variables used in construct validation, data on positive view of self was collected for the 15-year-olds in the project but not for the younger age cohorts. Therefore, validation was conducted only for the 15-year-olds. The following three measures were used [12]. Mental well-being was assessed using the short version of the Warwick–Edinburgh Mental Well-Being Scale (SWEMWBS). This short scale consists of seven items, such as ‘I’ve been feeling optimistic about the future’. Response options range from 1=‘never’ to 4=‘all the time’. Alpha reliability was 0.91. Self-esteem was measured on a scale based on Rosenberg’s general self-esteem model. Three items, such as ‘I like myself’, were used, with response options ranging from 1=‘strongly disagree’ to 5=‘strongly agree’. The three-item scale had an alpha reliability of 0.89. The scale for General self-efficacy consisted of two items: ‘How often do you find a solution to a problem if you try hard enough?’ and ‘How often do you manage to do things that you decide to do?’ The response options ranged from 1=‘never’ to 4=‘always’. It was based on Schwartzer’s theoretical model. The correlation between the two items was 0.57.

Measures of different types of school-related factors, which in previous research have been associated with mental health among adolescents [14], were also analysed for a more thorough construct validation of the profiles. Liking school was measured by a single item: ‘How do you feel about school at present?’ The response options were 1=‘I like it a lot’, 2=‘I like it a bit’, 3=‘I don’t like it very much’ and 4=‘I don’t like it at all’. The response scale was coded reversely. Teacher support and classmate support were assessed using the three-item Teacher Support Scale and the three-item Classmate Support Scale [15]. On a scale ranging from 1=‘strongly disagree’ to 5=‘strongly agree’, adolescents indicated the extent to which they experienced their teachers/classmates as supportive. Examples questions include ‘I feel that my teachers accept me as I am’ and ‘The students in my class enjoy being together’. Alpha reliability was 0.83 for teacher support and 0.77 for classmate support.

Finally, the adolescents’ perceptions of social support from parents and peers were used in the cross-validation. For perceived social support, support from family and friends was measured using the family and friends subscales of the Multidimensional Scale of Perceived Social Support [16]. On a scale ranging from 1=‘strongly disagree’ to 7=’strongly agree’, the adolescents rated how strongly they felt supported by their families (four items) using items such as ‘My family really tries to help me’ and by their friends (four items) using items such as ‘My friend really tries to help me’. Alpha reliability was 0.94 for family support and 0.92 for friend support.

### Socio-demographic characteristics

Sex was coded as 0=‘male’ or 1=‘female’. Family structure was a measure of which categories of adults resided in the home most of the time, differentiating between living with both biological parents (1) and living in other family structures (0). Family affluence was measured using the Family Affluence Scale [17], which is an indicator of young people’s socio-economic status and comprises six items on material assets in the family. An example of the items used is: ‘Do you have your own bedroom?’

### Analytical methods

Cluster analysis was used to identify the naturally occurring individual patterns/profiles of symptoms and perceived health in the samples. Cluster measures were based on the HBSC symptoms/complaints scale and self-rated overall health. A factor analysis of the eight items in the HBSC/SCL produced one factor at each age. These factors were used in the cluster analyses alongside the single item on self-reported overall health. Both variables were standardised (mean=0 and standard deviation=1) at each age. First, we applied a hierarchical cluster analysis (Ward’s method) to identify the number of clusters. We set the lower explanatory limit at 67% of the total error sum of squares for the number of clusters selected [18]. Thereafter, we used non-hierarchical cluster analyses, K-means clustering, to arrive at the final cluster solution for each age group. The cluster solutions for the 11-, 13- and 15-year-olds were compared.

In the next set of analyses, we combined the three age cohorts. It was not possible to calculate differences in prevalence across the three ages because the age samples were not of equal size. For this reason, we constructed a new data set. The number of people in each of the three age groups (11, 13 and 15) with complete data for psychosomatic symptoms and perceived overall health were 1074, 1346 and 1580, respectively. We selected the 1074 participants in the age-11 sample and randomly selected 1074 people in the age-13 and age-15 samples. A cluster analysis, using the guidelines as reported above, was performed.

To cross-validate the cluster groups, we used data on the 15-year-olds. The complete set of comparison variables was only available for this age group. The cross-validation was performed using measures of perceptions of a positive self, school experiences and perceived social support from parents and friends. A factor analysis of the measures generated three factors. Factor loadings ranged from 0.77 to 0.98 for the three measures of a positive self, from 0.72 to 0.84 for the three measures of good school experiences and from 0.83 to 0.87 for the two measures of social support from family and friends. Analyses of variance comparing the cluster groups for these three factors and measures of socio-demographic conditions (family structure and family affluence) were applied, and the effect sizes were estimated with η^2^.

In all cross-tabulations, we used the EXACON program, which examines single-cell frequencies in contingency tables [19]. A Bonferroni-adjusted p-value of .05 was used to determine which specific cells in the contingency table occurred more often (a Type) and less often (an Antitype) than expected by chance.

## Results

Table I reports on the cluster analyses conducted separately for the three age groups: 11-, 13- and 15-year-olds. Four clusters were found in all three age groups. We had expected to find mentally unhealthy adolescents in two clusters, and they were found in all three age groups: high levels of psychosomatic symptoms only (the high symptoms group) and high levels of psychosomatic symptoms and perceived poor overall health (poor mental health). In addition, at all ages, one cluster contained adolescents with high levels of perceived overall health (perceived good health). At ages 13 and 15, one cluster contained adolescents with average levels of both symptoms and perceived health (average). In contrast, the final cluster of the 11-year-olds contained adolescents with low perceived overall health (perceived poor health).

**Table I.** Cluster analyses of 11-, 13- and 15-year-old adolescents’ self-rated psychosomatic symptoms and perceived overall health.

11-year-olds	Perceived good health	Perceived poor health	High symptoms	Poor mental health
Psychosomatic symptoms	−0.46	−0.35	1.48	1.00
Perceived overall health	0.93	−0.83	−0.43	−2.68
*n*	506	337	207	35
%	47	31	19	3
Boys %	49	33	15^a^	3
Girls %	44	29	23^t^	4
13-year-olds	Perceived good health	Average	High symptoms	Poor mental health
Psychosomatic symptoms	−0.56	−0.48	1.20	1.31
Perceived overall health	1.05	−0.55	−0.27	−2.33
*n*	508	442	328	85
%	37	32	24	6
Boys %	43^t^	37^t^	16^a^	3^a^
Girls %	32^a^	27^a^	32^t^	9^t^
15-year-olds	Perceived good health	Average	High symptoms	Poor mental health
Psychosomatic symptoms	−0.60	−0.56	1.02	1.11
Perceived overall health	1.19	−0.44	−0.22	−2.15
*n*	502	532	449	129
%	31	33	28	8
Boys %	41^t^	39^t^	15^a^	5^a^
Girls %	23^a^	28^a^	39^t^	11^t^

Tests of sex differences used the EXACON program. Guidance for interpretation of psychosomatic symptoms and perceived overall health: a low value is <−0.70, an average value is between −0.70 and 0.70, a high value is >0.70 (roughly 24%, 47% and 24% of the sample).

Sex differences:

Age 11: χ^2^ (3 df)=11.44, *p*=0.010, Cramer’s V=0.10.

Age 13: χ^2^ (3 df)=72.23, *p*<0.001, Cramer’s V=0.23.

Age 15: χ^2^ (3 df)=155.10, *p*<0.001, Cramer’s V=0.30.

Perceived good health: high levels of overall health and average levels of psychosomatic symptoms; average: average levels of psychosomatic symptoms as well as overall health; high symptoms: high levels of psychosomatic symptoms and average levels of overall health; poor mental health: high level of psychosomatic symptoms and perceived poor overall health. EXACON: t: type (cell frequency is higher than expected by chance); a: antitype (cell frequency is lower than expected by chance).

Considering first the two clusters expected to have poorer mental health, the cluster with both high levels of psychosomatic symptoms and poor perceived overall health (poor mental health) was the smallest at all ages. Having a high level of psychosomatic symptoms only (high symptoms) was more common. There were between three and six times as many adolescents in the high symptoms cluster as in the poor mental health cluster.

At ages 13 and 15, EXACON analyses showed that girls were less likely than expected by chance (an antitype) to appear in the perceived good health and the average clusters and were more likely than expected by chance (type) to appear in the high symptoms and poor mental health clusters. The opposite applied to boys. The effect sizes were moderate. In short, girls dominated in the two clusters indicating poorer mental health. Fewer sex differences appeared at age 11, where the only significant pattern was in the high symptoms cluster. Girls were more likely than boys to report high levels of psychosomatic symptoms. The effect size was weak.

Overall, the general structure of the clusters for the 13-year-olds was very similar to the cluster for the 15-year-olds. It is the cluster structure for the 11-year-olds that stands out. First, there was no average cluster at age 11. Instead, the cluster analysis identified a perceived poor health cluster. Second, in comparison with ages 13 and 15, the sex differences for the 11-year-olds were small, with about as high a percentage of boys as girls (3% versus 4%) belonging to the poor mental health cluster.

The three age groups were merged into one sample (*N*=1074 each age). A common cluster analysis with the same centroid for each age group resulted in the four clusters reported in Table II (perceived good health, average, high symptoms and poor mental health). A cross-tabulation of age cohort and clusters showed that there were significant differences between the three ages (χ^2^=114.44, *p*<0.001, contingency coefficient=0.19). A subsequent EXACON analysis showed that the 11-year-olds were overrepresented in the perceived good health cluster and underrepresented among the participants in the high symptoms and poor mental health clusters. The opposite was the case for the 15-year-olds, that is, a decrease across ages in the proportion of people belonging to the perceived good health cluster and an increase in the proportions belonging to the high symptoms and poor mental health clusters.

**Table II.** Cluster analysis of psychosomatic symptoms and perceived overall health for the three ages combined.

	Perceived good health	Average	High symptoms	Poor mental health
Psychosomatic symptoms	−0.56	−0.48	1.18	1.28
Perceived overall health	1.05	−0.60	−0.33	−2.39
*n*	1237	998	806	181
%	38	31	25	6
% age 11	47^t^	32	18^a^	3^a^
% age 13	38	32	24	6
% age 15	30^a^	29	33^t^	8^t^

Guidance for interpretation of psychosomatic symptoms and perceived overall health: a low value is <−0.70, an average value is between −0.70 and 0.70 and a high value is >0.70.

Test of age differences using the EXACON program: t: type (cell frequency is higher than expected by chance); a: antitype (cell frequency is lower than expected by chance).

### Construct validity

We used the factor scores of a perceived positive self, positive school experiences and perceived social support from family and friends to construct validate the four clusters. They were measured only among the 15-year-olds. We expected that the cluster groups would emerge in the following order for each of the mental-health profiles: poor mental health<high symptoms<average<perceived good health. Differences between the four clusters are reported in Table III, and all *F*-values were significant at the 0.001 level. The adolescents in the perceived good health cluster consistently had the highest values on the positive health indicators, and the adolescents in the poor mental health cluster consistently had the lowest. They followed the expected order for all mental-health profiles. The effect size was large for positive self, medium for positive school experiences and low for social support from family and friends. The findings, especially for positive self and school experiences, provide support for the construct validity of the four clusters. Finally, although there were significant differences among the four mental health profiles for the two socio-demographic indicators (family structure and family affluence), the effect sizes were low.

**Table III.** Cross-validation of cluster profiles among 15-year-olds in relation to measures of positive self, school satisfaction and others’ support, and comparisons for two socio-demographic conditions.

	Perceived good health (*F*-values)	Average	High symptoms	Poor mental health
Positive self	0.61^d^153.24	−0.02^c^ 0.24	−0.43^b^	−0.91^a^
Positive school experiences	0.32^d^ 58.55	0.09^c^ 0.11	−0.28^b^	−0.78^a^
Others’ support	0.22^c^ 16.00	0.00^b^ 0.03	−0.12^b^	−0.38^a^
Family structure	0.18^b^ 8.65	−0.02^a^ 0.02	−0.11^a^	−0.19^a^
Family affluence	0.24^b^ 14.18	−0.13^a^ 0.03	0.00^a^	−0.17^a^

All measures in the table are standardised. Across rows, superscripts represent significant differences (*p*<0.05) among the four clusters in Student–Neuman–Keuls post hoc tests. All *F*-values are significant at the 0.001 level. η^2^ is used as a measure of effect size: η^2^=0.01 indicates a small effect; η^2^=0.06 indicates a medium effect; η^2^=0.14 indicates a large effect.

Positive self: self-esteem, general self-efficacy, Warwick–Edinburgh Mental Well-Being Scale; positive school experiences: support of teachers, support of classmates, liking school; others’ support: social support of family and friends.

## Discussion

The current study provides further evidence supporting a dual-factor model of mental health. The findings yielded four distinct groups based on differing levels of perceived overall health and psychosomatic complaints: perceived good health, average health (perceived poor health in the age-11 sample), high psychosomatic symptoms and poor mental health. The main novelty of our research was its generation of mental-health profiles in large representative adolescent samples comprising three age groups. Theoretically defined cut-offs on two dimensions always give four groups, but an empirical analysis is open to different solutions. The presence of these four unique groups indicates that degrees of mental health are not simply the opposite of degrees of psychopathology, and that it is worthwhile to base an analysis on indicators of perceived overall health and psychosomatic complaints. In this study, the correlation between psychosomatic symptoms and overall health was −0.38 (*p*<0.001). The magnitude of this correlation is consistent with previous studies demonstrating that psychological distress and well-being are related but still distinct domains in mental health [5,20].

Descriptions of mental health among adolescents have often been based on psychosomatic problems, but poor mental health does not necessarily equate to a high level of psychosomatic problems. It was possible in this study to identify a cluster of adolescents with a high level of psychosomatic symptoms only. In all cross-validation analyses, they had better health and adjustment than the adolescents in the cluster group reporting both a high level of psychosomatic symptoms and a low level of perceived overall health.

Analyses of differences between the three age groups (11-, 13- and 15-year-olds) showed that the clusters for the 13- and 15-year-olds were very similar. The 11-year-olds stand out. First, the percentage of adolescents in the poor mental health cluster was lower among the 11-year-olds than among the older adolescents. Second, there were no sex differences in this cluster. The sex differences appeared in the older age groups. What was unique about the 11-year-olds was also that there was one cluster group that contained persons with poor perceived overall health but only average psychosomatic problems (the age-13 and age-15 clusters were average on these two cluster measures). Potentially, these might be adolescents who later also develop psychosomatic symptoms [21], especially poor adjustment. Without longitudinal data, we cannot examine trends over the ages for the same persons, but there may be data sets that would enable developmental trends in the mental health of adolescents who evaluate their overall health negatively even at an early age to be tracked.

The present study showed a large effect size when the relationships with indicators of a positive self were analysed across clusters and medium effects for positive school experiences. They attest to the construct validity for the identified mental-health profiles.

Much further research can be developed using the dual-factor model [5,22]. Mental health among adolescents has deteriorated, especially among girls. The model can be used to reveal different patterns of changes during the entire time span from 2002 to 2018 among boys and girls in different age groups in the HBSC surveys. The present study was partly based on the measure of perceived overall health. More qualitative and mixed-method approaches could enhance understanding of the different mental-health profiles.

Concerning the age differences analysed with the three age groups combined, there was a particularly strong increase across the ages in the proportion of participants in the high psychosomatic symptoms cluster (from 18% to 33% from age 11 to age 15) but a weaker increase in the poor mental health cluster (from 3% to 8%). As a consequence, there was a decrease in the proportion of participants in the perceived good health cluster (from 47% to 30%). For the average cluster (average levels of symptoms and perceived overall health), there were no significant differences across the ages (from 32% to 29%). Concerning sex differences in each of the three age groups, only one difference was significant at age 11. There was a higher proportion of girls in the high symptoms cluster. There were further sex differences in the age-13 and age-15 clusters. The proportions of girls were higher than those of boys in the poor mental health cluster and in the high symptoms cluster, and the proportions of girls were lower in the perceived good health cluster and average cluster. Hence, different types of mental-health problems seem to increase after the age of 11.

### Strengths and limitations

A major strength of the study is that it is based on large-scale data from a nationally representative sample of Swedish pupils aged 11, 13 and 15 (grades 5, 7 and 9). The measures used have good validity and reliability according to previous research [13–17,23]. Although the voices of the adolescents themselves are indispensable in research on adolescent mental health, the availability of independent sources of information – from parents, teachers, friends and others – would have strengthened our study. This would have offered the possibility, for example, to elucidate if other people around the adolescents recognise the differences in psychosocial adjustment between adolescents with high psychosomatic symptoms and adolescents with both psychosomatic symptoms and poor perceived overall health, and between adolescents with good perceived overall health and poor perceived overall health. Further, this would have offered the opportunity to find out if parents, teachers, friends and others base their judgements on the mental health of adolescents on their perceptions of the adolescents’ psychosomatic complaints and/or the adolescents’ overall evaluations of their health.

The cross-sectional design does not allow causal inferences. Hence, future longitudinal studies are needed [24]. The dual-factor model of mental health is increasingly supported [5], but the relevant studies are mostly cross-sectional. However, analyses of repeated cross-sectional studies can illuminate the added value of using person-centred analysis to describe adolescent mental health [25].

## Conclusions

The present study shows the utility of applying a person-centred approach to the study of adolescents’ mental-health problems. Apparently, adolescents’ reports of their psychosomatic complaints need to be integrated with their perceived overall health. Those adolescents with high levels of psychosomatic problems and perceptions of poor overall health make up the group of adolescents who show the poorest psychosocial adjustment. They need special consideration in treatment.
